# Dosimetric comparison of different treatment modalities for stereotactic radiotherapy

**DOI:** 10.1186/s13014-017-0890-0

**Published:** 2017-09-16

**Authors:** Shih-Ming Hsu, Yuan-Chun Lai, Chien-Chung Jeng, Chia-Ying Tseng

**Affiliations:** 1 Medical Physics and Radiation Measurements Laboratory, National Yang-Ming University, Taipei, Taiwan, ROC; 2 Department of Biomedical Imaging and Radiological Sciences, National Yang-Ming University, No. 155, Sec. 2, Li-Nong St., Beitou District, Taipei, 112 Taiwan, ROC; 3 Biophotonics and Molecular Imaging Research Center, National Yang-Ming University, Taipei, Taiwan, ROC; 4 Department of Physics, National Chung Hsing University, Taichung, Taiwan, ROC; 5 Department of Radiation Oncology, Changhua Christian Hospital, Changhua, Taiwan, ROC

**Keywords:** Linac, Stereotactic radiotherapy, Tomotherapy, Treatment planning systems, VMAT

## Abstract

**Background:**

The modalities for performing stereotactic radiotherapy (SRT) on the brain include the cone-based linear accelerator (linac), the flattening filter-free (FFF) volumetric modulated arc therapy (VMAT) linac, and tomotherapy. In this study, the cone-based linac, FFF-VMAT linac, and tomotherapy modalities were evaluated by measuring the differences in doses delivered during brain SRT and experimentally assessing the accuracy of the output radiation doses through clinical measurements.

**Methods:**

We employed a homemade acrylic dosimetry phantom representing the head, within which a thermoluminescent dosimeter (TLD) and radiochromic EBT3 film were installed. Using the conformity/gradient index (CGI) and Paddick methods, the quality of the doses delivered by the various SRT modalities was evaluated. The quality indicators included the uniformity, conformity, and gradient indices. TLDs and EBT3 films were used to experimentally assess the accuracy of the SRT dose output.

**Results:**

The dose homogeneity indices of all the treatment modalities were lower than 1.25. The cone-based linac had the best conformity for all tumors, regardless of the tumor location and size, followed by the FFF-VMAT linac; tomography was the worst-performing treatment modality in this regard. The cone-based linac had the best gradient, regardless of the tumor location and size, whereas the FFF-VMAT linac had a better gradient than tomotherapy for a large tumor diameter (28 mm). The TLD and EBT3 measurements of the dose at the center of tumors indicated that the average difference between the measurements and the calculated dose was generally less than 4%. When the 3% 3-mm gamma passing rate metric was used, the average passing rates of all three treatment modalities exceeded 98%.

**Conclusions:**

Regarding the dose, the cone-based linac had the best conformity and steepest dose gradient for tumors of different sizes and distances from the brainstem. The results of this study suggest that SRT should be performed using the cone-based linac on tumors that require treatment plans with a steep dose gradient, even as the tumor is slightly irregular, we should also consider using a high dose gradient of the cone base to treat and protect the normal tissue. If normal tissues require special protection exist at positions that are superior or inferior to the tumor, we can consider using tomotherapy or Cone base with couch at 0° for treatment.

## Background

Stereotactic radiosurgery (SRS) can be used to treat arteriovenous malformation, glioblastoma multiforme, and various metastasized tumors in the brain. Because lesions that are treated using SRS tend to be very small, and the method in which the doses are delivered differs from the traditional multi-fractionated dose-delivery mode, the required radiation in SRS is usually delivered in a single dose. In stereotactic radiotherapy (SRT), the required dose is fractionated into multiple doses. Because each of the doses in a single SRT or SRS treatment is extremely large, a high accuracy is necessary for the treatments, as well as a very steep dose gradient, to ensure the tumor is given a sufficiently high dose while the surrounding normal tissues are protected and left unharmed [1]. In recent years, advancements in linear accelerator (linac) based technologies, including developments in image-guidance systems, multileaf collimators (MLCs), and volumetric-modulated arc therapy (VMAT), have led to linac-based treatments that can achieve a high accuracy, steep gradients, and a high level of conformity [2–9].

Traditionally, the Gamma Knife has been the primary tool for performing brain SRS or SRT. However, the sites without this facility instead utilize tomotherapy and MLC or cone-based linac devices to perform SRT therapy. Tomotherapy has a linac mounted on a ring gantry, and a binary MLC is used to adjust the dose of the photon-beam irradiation in sync with the forward motion of the treatment couch, resulting in a helical and tomographic form of intensity modulated radiotherapy (TomoHelical IMRT). Linear accelerators may use either an MLC or a cone to shape and limit the field of radiation. Recent developments in flattening filter-free (FFF) high-dose models have led to a further reduction in the probability of patient movement, thus reducing the effects caused by patient movement. This has enabled the FFF-VMAT linac treatment modality to become a viable tool for performing SRT.

Different treatment modalities have different output dose characteristics, which may affect the radiation doses received by normal tissues surrounding the tumor [10–12]. Therefore, the main goal of this study is to compare the SRT treatment doses of the cone-based linac, FFF-VMAT linac, and tomotherapy treatment modalities and evaluate the differences between doses calculated according to treatment planning systems and measured radiation doses.

## Methods

### Design of dosimetry phantom

**Fig. 1:**
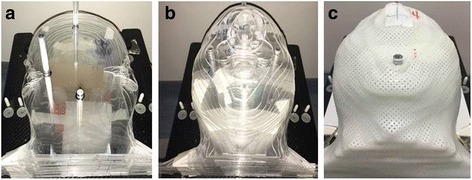
Homemade acrylic dosimetry phantom of the head: (**a**) coronal slices and fixing rods, (**b**) head phantom formed from all of the coronal slices, and (**c**) head phantom fixed by a mask

A homemade acrylic phantom of the head was created according to computed-tomography (CT) images of patients. EBT3 radiochromic films (Ashland, USA) and the cubic thermoluminescent dosimeters (Thermo, USA) were installed within the coronal slices to measure the distribution of the radiation doses. In the region where the tumor was located the slices were 2 mm thick; the thickness of the remaining slices was 5 mm, as shown in Fig. 1.

### Treatment planning

**Fig. 2:**
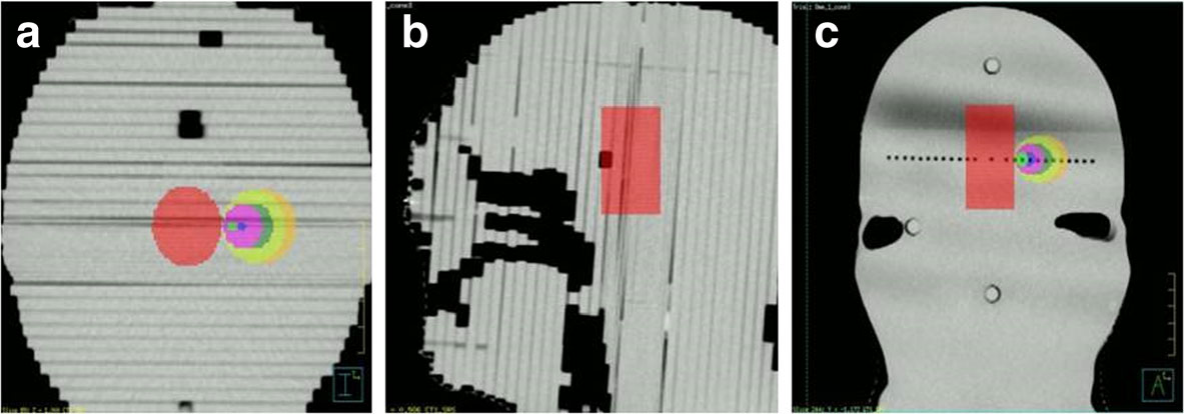
CT images of the homemade acrylic dosimetry phantom of the head: (**a**) transverse view, (**b**) sagittal view, (**c**) coronal view (Red, the simulated brainstem; Green, tumor of 8 mm diameter and 1 mm away from brainstem; Blue, tumor of 8 mm diameter and 6 mm away from brainstem; Pink, tumor of 18 mm diameter and 1 mm away from brainstem; Dark-green, tumor of 18 mm diameter and 6 mm away from brainstem; Yellow-green, tumor of 28 mm diameter and 1 mm away from brainstem; Orange, tumor of 28 mm diameter and 6 mm away from brainstem.)

CT (LightSpeed GE, USA) was applied to obtain images of the phantom, and the thickness of each CT slice was 1.25 mm. The images were sent to a tomotherapy treatment planning system (TomoTherapy Planning Station Hi-ART® version 4.3.2 Accuray, USA) and a Pinnacle treatment planning system (Pinnacle^3^® Version 9.8 Philips, USA). The TomoTherapy system designed all the planning related to tomotherapy, and the Pinnacle system designed all the planning for the cone-based and FFF-VMAT linacs. All necessary beam data were entered into the Pinnacle system, and commission tests were completed for this system. A structure with a diameter of 3 cm was placed on the CT images to simulate the location of the brainstem. To match the size of the collimating cone, spherical tumors 8, 18, and 28 mm in diameter were placed at distances of 1 and 6 mm from the borders of the brainstem, as shown in Fig. 2. The use of spherical tumors is expected to exclude the effects of tumor shapes in order to purely assess the differences in different modalities.

**Fig. 3:**
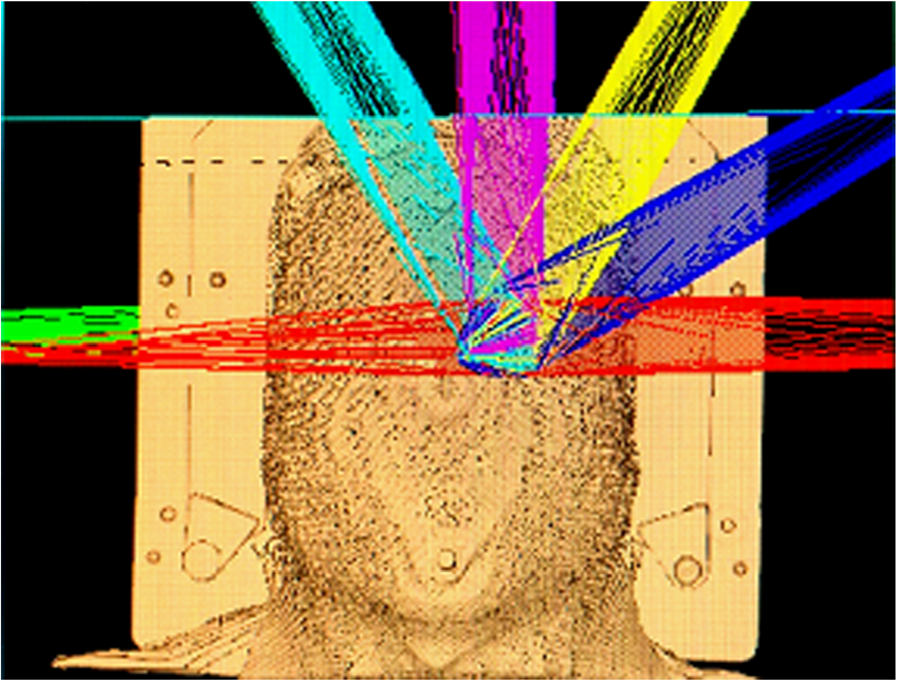
All the beam angles used by the Pinnacle treatment planning system

Different treatment plans were designed by the treatment planning systems according to the size of the tumors. To exclude the effects of the beam angle, all the cone-based linac and FFF-VMAT linac plans used the arc-therapy method and the same beam angles, as shown in Fig. 3. The beam angles were as follows: counter-clockwise from 179° to 345° with a collimator angle of 0° and a couch angle of 0°, counter-clockwise from 210° to 180° with a collimator angle of 10° and a couch angle of 0°, counter-clockwise from 179° to 0° with a collimator angle of 330° and a couch angle of 330°, counter-clockwise from 179° to 0° with a collimator angle of 310° and a couch angle of 300°, counter-clockwise from 179° to 0° with a collimator angle of 260° and a couch angle of 270°, and clockwise from 180° to 0° with a collimator angle of 250° and a couch angle of 60°.

The accelerator used for the cone-based linac was a 6-MV Elekta Synergy (Sweden) photon. Cone diameters of 10, 20, and 30 mm were used. The accelerator used for the FFF-VMAT linac was a 6-MV FFF Elekta Axesse (Sweden) photon beam and was used in conjunction with a 5 mm wide Elekta Agility high-speed MLC. The following parameters were used for the TomoTherapy system (Accuray, USA): modulation factor of 3.0, field width of 1 cm, pitch of 0.086.

All treatment planning were planned by the same planner. In all treatment plans, a dose of 600 cGy was delivered to the tumors, and the conditions were optimized to maintain 98% dose coverage on the tumor, with the maximum tumor dose not exceeding 125% to minimize the doses incident on the brainstem and normal brain tissue. Maximum dose constrain of brainstem for tumor away from brainstem 1 mm and 6 mm were limited to 500 cGy and 200 cGy, respectively and D_1%_ of these were limited to 150 cGy and 100 cGy, respectively. Auxiliary ROI_s_ such as “Ring” structure were also used to decrease the dose of normal tissue during planning. The three-dimensional (3D) dose grid of the three axes of the Pinnacle system was set to 1 mm and that of the tomotherapy system was set to “fine”.

### Analytic indicators of dose quality

The homogeneity index (HI) [13], conformity/gradient index (CGI), and Paddick indices were used to compare the quality of the treatment plans. The HI was used to describe the homogeneity of the dose within the tumor. The HI is defined as

1 $$ \mathrm{HI}=\mathrm{MD}/\mathrm{PD}, $$

where MD is the maximum dose within the tumor, and PD is the 100% prescription dose. The HI of a perfect treatment plan is 1; if the 80% isodose curve is selected as the prescription dose, then the HI becomes 1.25 instead.

The CGI is a holistic index that consists of the conformity index (CGI_c_) and the gradient index (CGI_g_) [14]. The CGI is defined as

2 $$ CGI=\left({CGI}_C+{CGI}_g\right)/2 $$

The CGI_c_ is used to describe the relationship between the volume of the tumor and the volume covered by the dose. The CGI_c_ is defined as

3 $$ {CGI}_C=\left(\frac{TV}{PIV}\right)\times 100 $$

PIV is the volume covered by the 100% prescription dose curve, and TV is the volume of the tumor. CGI_c_ = 100 corresponds to perfect conformity of the treatment planning. .

The CGI_g_ is the effective difference in radius between the volumes covered by the 50% and 100% doses; it is used to evaluate the decrement of the dose in the high-dose region (50% and above) and is defined as follows:

4 $$ {CGI}_g=100-\left\{100\times \left[\left({R}_{eff,50\% Rx}-{R}_{eff, Rx}\right)-0.3 cm\right]\right\} $$

*R*_*eff* , *Rx*_ refers to the effective radius of the volume covered by the 100% prescription dose curve, and *R*_*eff* , 50 % *Rx*_ is the effective radius of the volume covered by the 50% prescription dose curve, with *R*_*eff*_ defined as

5 $$ {R}_{eff}=\sqrt[3]{\frac{3V}{4\pi }} $$

V is the volume covered by the required dose. This 3 mm in distance between *R*_*eff* , 50 % *Rx*_ and *R*_*eff* , *Rx*_ gradient was obtained empirically from clinical radiosurgery planning cases, and corresponds to the possible gradient with linac radiosurgery when using multiple noncoplanar arcs. As CGI_g_ more than 100, it corresponds to less gradient than an optimum 3 mm empirically..

The Paddick indices are clinically used to describe the conformity [15] and gradient [16] of the treatment plan. The Paddick indices include the conformity index (CI_Paddick_) and the gradient index (GI_Paddick_). As the CGI_c_ is unable to present the degree of tumor-volume coverage for a specified prescription dose curve, the CI_Paddick_ complements the CGI_c_ by describing the volume covered by the prescription dose as well as the relationship between the tumor volume covered by the prescription dose and the overall volume of the tumor. The CI_Paddick_ is defined as

6 $$ {\mathrm{CI}}_{Paddick}=\frac{{\left({TV}_{PIV}\right)}^2}{TV\times PIV} $$

where PIV refers to the volume covered by the 100% prescription dose curve, TV is the tumor volume, and TVPIV is the tumor volume covered by PIV. This index represents the degree to which a tumor is covered by a specified isodose curve. For a perfect treatment plan, CI_Paddick_ = 1.

The GI_Paddick_ describes the decrement of the dose in the high-dose region (50% and above) and is defined as

7 $$ {GI}_{Paddick}=\frac{V_{50\%}}{V_{100}} $$

where V50% is the volume covered by 50% of the prescription dose, and V100% is the volume covered by 100% of the prescription dose. The GI_Paddick_ can represent the degree to which normal tissues outside the tumor are protected. A perfect treatment plan must have a value of the GI_Paddick_ that approaches 1.

### Radiation dose measurements

In this work, cubic TLD-100 dosimeters and EBT3 films were used to measure the radiation dose [17–22], with the dose at each tumor location measured three times. Each cone-based linac and FFF-VMAT linac measurement was accompanied by alignment using the six degree-of-freedom image-guided cone-beam CT of the accelerator. MVCT was used to align the tomotherapy measurements to correct for 3D position shifts and roll-angle deviations in the rotating gantry. FILM QATM Version 2.2 was used to evaluate the profile changes in the right–left (R–L) and superior–inferior (S–I) directions, and gamma evaluation was used to determine the differences between the calculated dose of the treatment plan and the two-dimensional planar dose measured by the EBT3 films. Because SRT treatments characteristically require a high level of positional accuracy and a steep gradient, the 3% 3-mm and 5% 1-mm criteria were chosen as the gamma passing rate metrics for assessing the differences in the planar doses.

### Statistical analysis

The Mann–Whitney test (Statistical Package for the Social Sciences, IBM Corporation, New York, USA) was used to assess the statistical significance of the gamma analysis results for the different techniques.

## Results

### Dose-quality analysis

The results of the cone-based linac, FFF-VMAT linac, and tomotherapy dose-quality analyses are shown in Table 1. The average HI values of the cone-based linac, the FFF-VMAT linac, and tomotherapy were 1.20 ± 0.03, 1.21 ± 0.03, and 1.23 ± 0.02, respectively. As it was strictly specified that the maximum dose on the tumors did not exceed the 125% of the prescription dose during the creation of the treatment plans, none of the average HIs of the treatment plans exceeded 1.25. The lowest HI (1.16) was observed when the cone-based linac was used to treat 28 mm diameter tumors that were located 6 mm from the brainstem.

**Table 1:** HI, CGI, and Paddick indices calculated by the treatment planning system using the cone-based linac, FFF-VMAT linac, and tomotherapy treatment modalities

Tumor Diameter	8 mm						18 mm						28 mm					
Distance from brainstem	1 mm			6 mm			1 mm			6 mm			1 mm			6 mm		
Modality	Cone-based	FFF- VMAT	Tomo	Cone-based	FFF- VMAT	Tomo	Cone-based	FFF- VMAT	Tomo	Cone-based	FFF- VMAT	Tomo	Cone-based	FFF- VMAT	Tomo	Cone-based	FFF- VMAT	Tomo
HI	1.24	1.25	1.20	1.23	1.25	1.23	1.20	1.19	1.25	1.20	1.17	1.24	1.17	1.23	1.21	1.16	1.19	1.23
CGI_c_	84.59	48.89	47.96	86.25	67.93	46.79	92.02	75.66	69.75	95.35	73.99	67.76	98.99	95.44	86.04	99.84	92.27	86.68
CGI_g_	103.25	66.45	74.39	103.15	74.30	75.96	88.43	60.53	60.61	89.33	59.71	62.68	74.02	52.79	48.02	75.26	61.62	50.49
CGI	93.92	57.67	61.18	94.71	71.11	61.38	90.23	68.10	65.18	92.34	66.85	62.22	86.50	74.11	67.03	87.55	76.95	68.58
CI_Paddick_	0.82	0.48	0.47	0.84	0.66	0.45	0.90	0.73	0.68	0.92	0.72	0.66	0.94	0.92	0.84	0.95	0.88	0.81
GI _Paddick_	4.23	10.97	9.07	4.28	10.58	8.57	3.02	4.90	4.83	3.00	4.93	4.62	2.73	3.67	4.14	2.69	3.21	3.90

The average CGI values for the cone-based linac, the FFF-VMAT linac, and tomotherapy were 90.88 ± 3.37, 69.13 ± 6.74, and 64.26 ± 3.13, respectively. In general, the FFF-VMAT linac had higher CGI values than tomotherapy except for tumors with small distance from the brainstem. The average CGI_c_ values for the cone-based linac, the FFF-VMAT linac, and tomotherapy were 92.84 ± 6.41, 75.70 ± 17.01, and 67.50 ± 17.48, respectively. For all distances from the brainstem and tumor diameters, the cone-based linac exhibited the highest CGI_c_ values, followed by the FFF-VMAT linac, and finally tomotherapy.

The average CGI_g_ values for the cone-based linac, the FFF-VMAT linac, and tomotherapy were 88.91 ± 12.78, 62.57 ± 7.23, and 62.03 ± 11.65, respectively. The FFF-VMAT linac had higher CGI_g_ values than tomotherapy for large-diameter tumors (28 mm), with ratios of 52.79: 48.02 and 61.62: 50.49 for distances of 1 and 6 mm from the brainstem, respectively. The average CI_Paddick_ for the cone-based linac, the FFF-VMAT linac, and tomotherapy were 0.90 ± 0.05, 0.73 ± 0.16, and 0.65 ± 0.16, respectively. Regardless of the tumor diameter and distance from the brainstem, the cone-based linac exhibited the best CI_Paddick_ values, followed by the FFF-VMAT linac, and finally tomotherapy. The GI_Paddick_ value of the FFF-VMAT linac for large-diameter tumors (28 mm) was better than that of tomotherapy, with ratios of 3.67: 4.14 and 3.21: 3.90 for distances of 1 and 6 mm from the brainstem. However, tomotherapy had better GI_Paddick_ values than the FFF-VMAT linac for smaller-diameter tumors (8 mm), with ratios of 9.07: 10.97 and 8.57: 10.58 for distances of 1 and 6 mm from the brainstem, respectively.

### Dose measurements

**Fig. 4:**
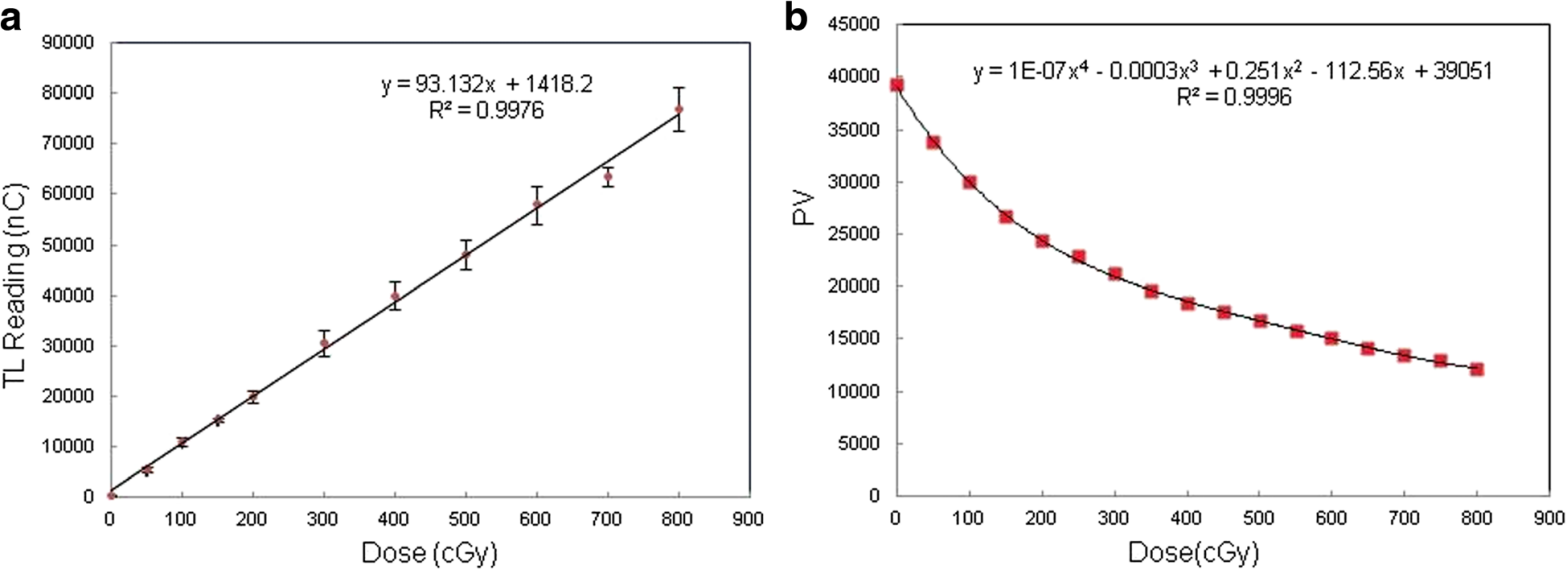
Dose-linearity curves for 6 MV: (**a**) TLD and (**b**) EBT3

The dose-linearity curves of TLD and EBT3 (0–800 cGy) are shown in Fig. 4. The radiation doses at the center of the tumors within the phantom were clinically measured and compared with the dose calculated in the treatment plan; the differences are shown in Table 2. The TLD and EBT3 dose measurements indicated that the average differences between the output doses of the cone-based linac, the FFF-VMAT linac, and tomotherapy and the calculated doses of the treatment plans were generally lower than 4%. The largest observed difference was −3.68% (−4.52% to −3.27%) for the cone-based linac and was measured using the EBT3 film.

**Table 2:** Average difference in the dose at the center of tumors between the calculated doses from the treatment planning system and the doses measured using TLD and EBT3 for the cone-based linac, the FFF-VMAT linac, and tomotherapy

Dosimeter	Cone-based linac	FFF-VMAT linac	Tomotherapy
TLD (%)	−1.38 (−4.08 to 0.81)	−1.34 (−4.96 to 4.04)	1.05 (−2.10 to 3.65)
EBT3 (%)	−3.68 (−4.52 to −3.27)	−1.75 (−4.70 to 2.52)	−1.02 (−3.72 to 0.58)

**Fig. 5:**
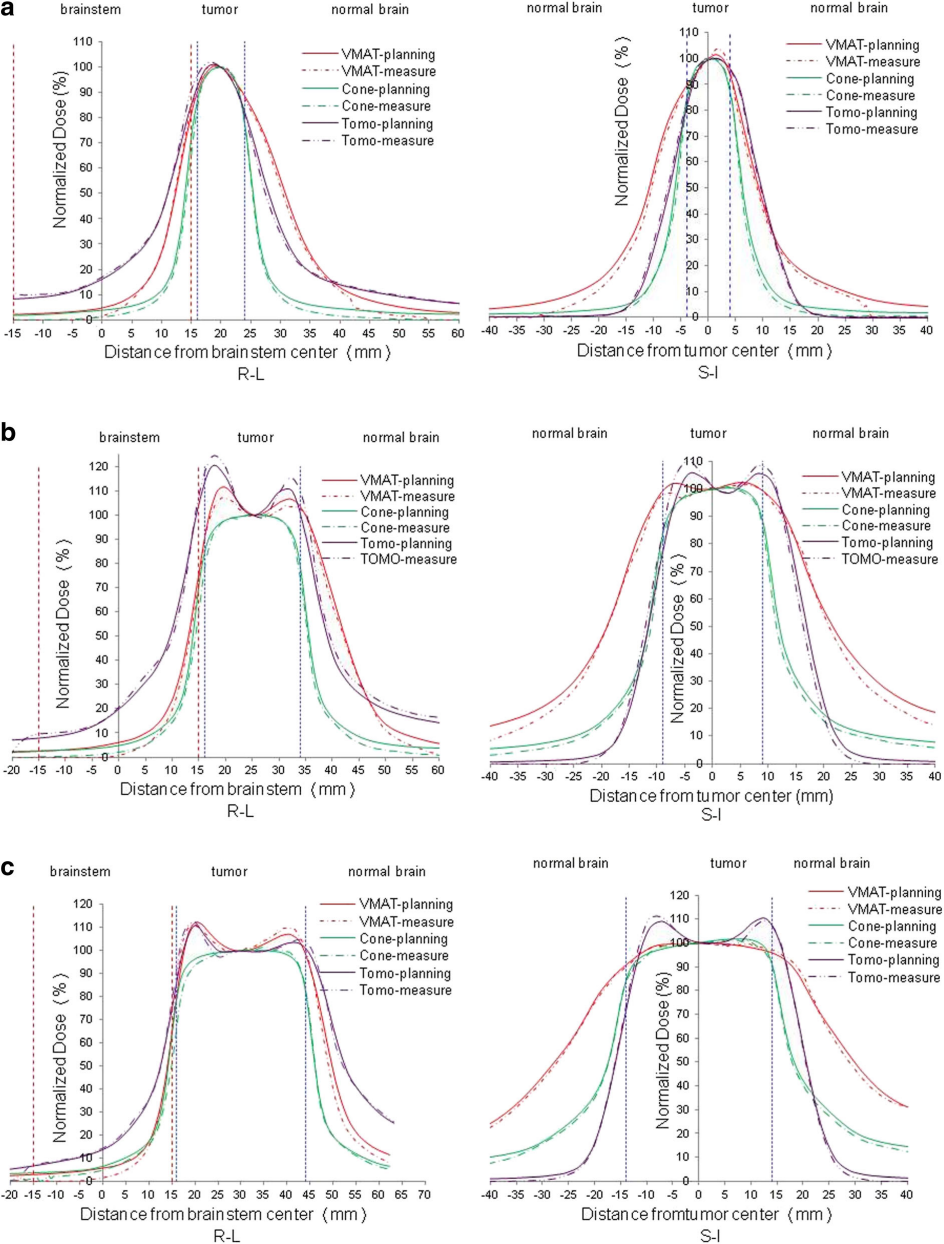
Differences of EBT3-measured and treatment plan-calculated profiles (normalized to the dose of tumor center) in the R–L and S–I directions for tumors located 1 mm from the brainstem with diameters of (**a**) 8 mm, (**b**) 18 mm, and (**c**) 28 mm

**Fig. 6:**
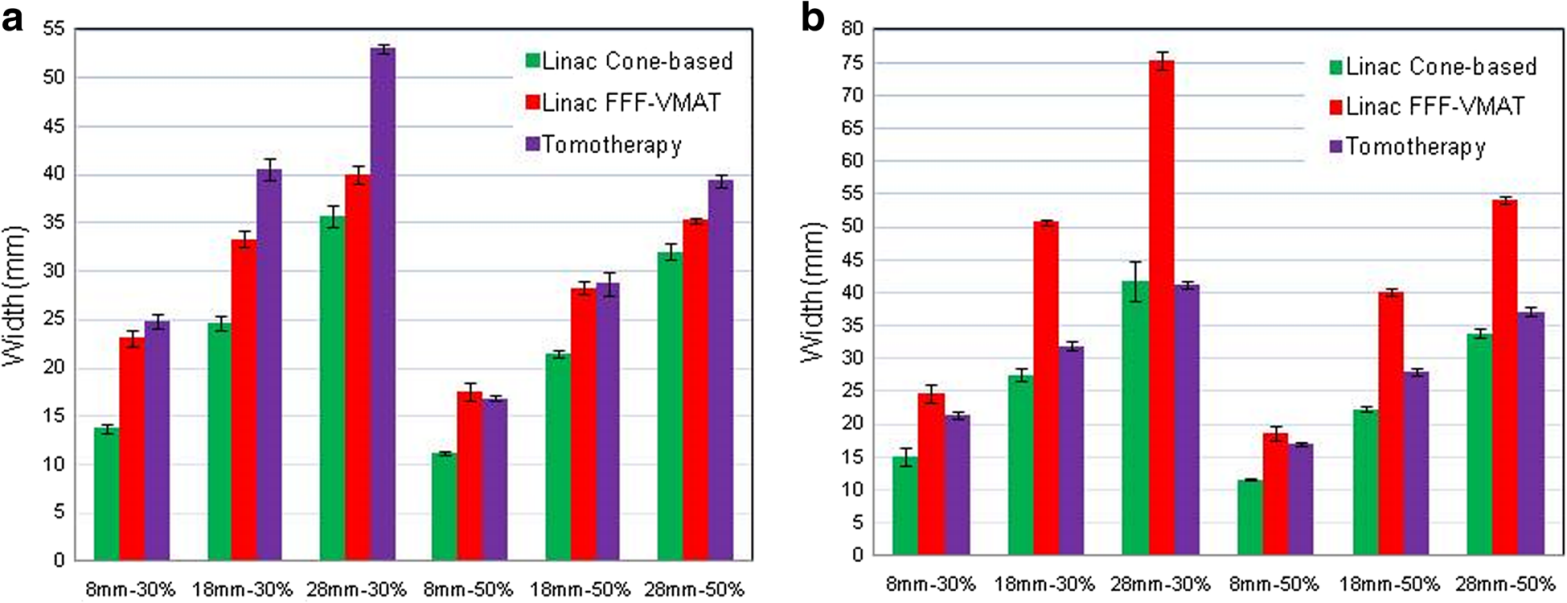
Average dose profile widths of doses that are 50% and 30% of the dose at the center of the tumor, measured using EBT3 films, in the (**a**) R–L direction and (**b**) S–I direction

Figure 5 shows the dose profile measured using EBT3 films for tumors locaed 1 mm from the brainstem in the R–L and S–I directions. The results of the measurement using the EBT3 film are generally consistent with calculations of the treatment plan. The average dose profile widths of the 50% and 30% doses relative to the dose at the center of the tumor for tumors of various sizes are shown in Fig. 6. The 30% profile widths of the FFF-VMAT linac were smaller than those of tomotherapy for all tumor volumes because the gantry rotated in a coplanar fashion, synchronized with the movement of the treatment couch, during the tomotherapy treatments to enable irradiation of the tumor. Because the R–L direction lies within the beam pathway, the distribution of low-intensity doses in the R–L direction was broadened.

**Fig. 7:**
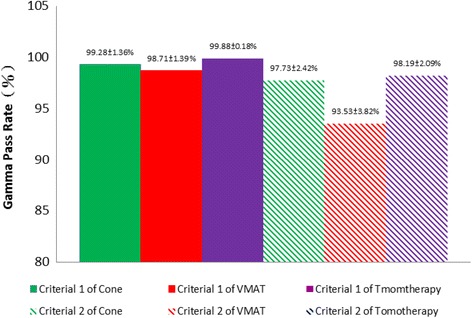
Gamma passing rates of the three treatment modalities under two different gamma passing criteria: Criteria 1: 3% 3-mm and Criteria 2: 5% 1-mm

The gamma passing rates of the three treatment modalities under two different gamma passing criteria are shown in Fig. 7. When the 3% 3-mm criteria was used as the passing metric, the passing rates for the cone-based linac, the FFF-VMAT linac, and tomotherapy were 99.28 ± 1.36%, 98.71 ± 1.39%, and 99.98 ± 0.18%, respectively. Among these, tomotherapy had the highest passing rate (*p* < 0.05), and there was no statistically significant difference between the passing rates for the cone-based and FFF-VMAT linacs (*p* = 0.235). Considering that SRT treatments characteristically require a high level of positional accuracy and steep dose gradients, the treatment modalities were also evaluated using the 5% 1-mm criteria as the passing metric. In this case, the passing rates for the cone-based linac, the FFF-VMAT linac, and tomotherapy were 97.73 ± 2.42%, 93.53 ± 3.82%, and 98.19 ± 2.09%, respectively. Among these, the FFF-VMAT linac had the lowest passing rate (*p* < 0.01), and there was no significant difference between the passing rates for the cone-based linac and tomotherapy (*p* = 0.46).

**Fig. 8:**
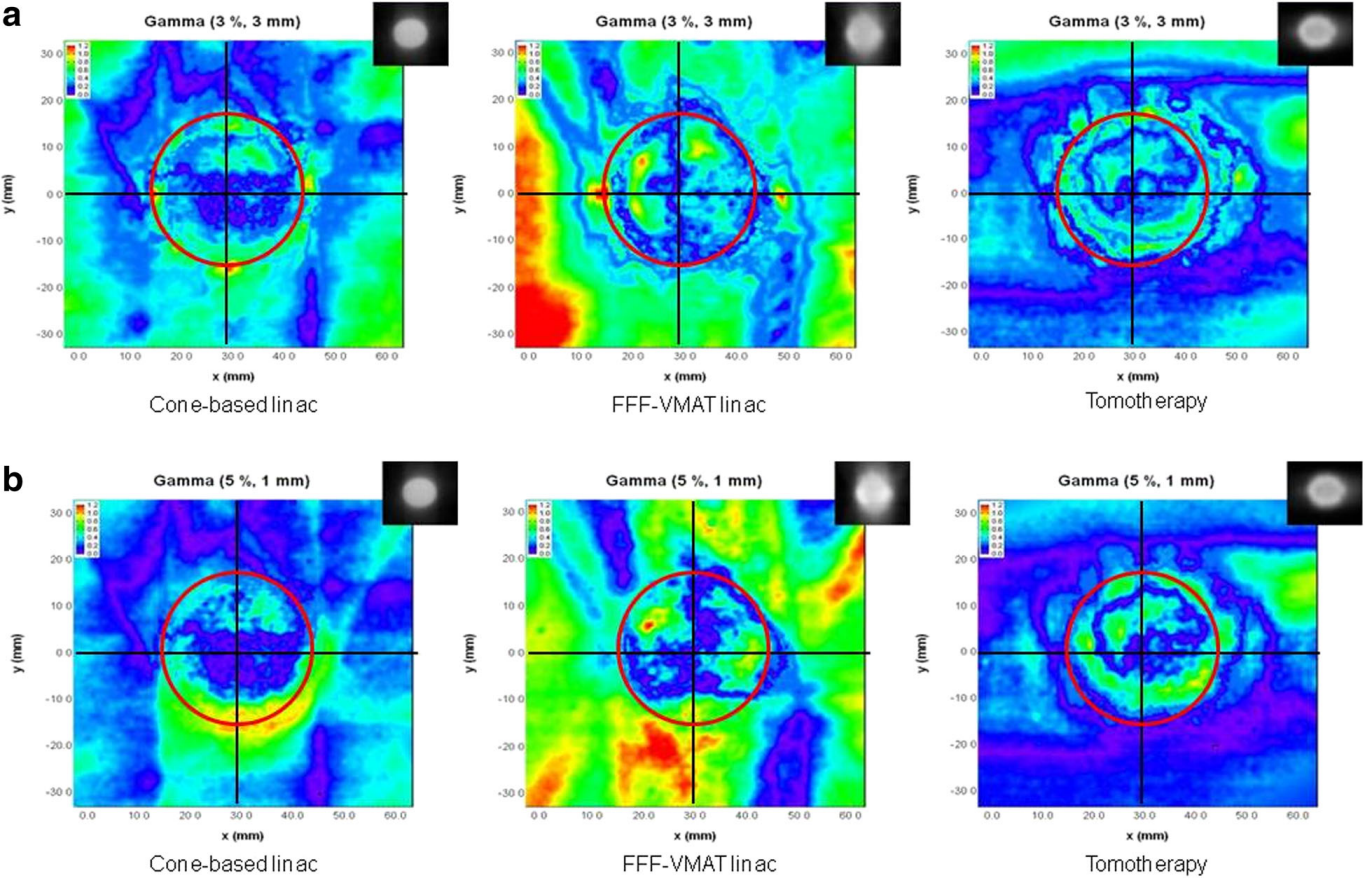
Gamma-evaluation maps of the three different treatment modalities evaluated using two different gamma passing criteria (the red circle indicates the area of the 28 mm tumor that is located 1 mm from the brainstem): (**a**) 3% 3-mm and (**b**) 5% 1-mm

The gamma evaluation maps of 28 mm diameter tumors located 1 mm from the brainstem using the 3% 3-mm and 5% 1-mm criteria were analyzed, as shown in Fig. 8. Here, changing the passing metric caused an increase in the failed areas in regions with steep doses at the borders of the tumor for the cone-based and FFF-VMAT linac modalities. The cone-based linac and tomotherapy retained a gamma passing rate of 95% and above, whereas the passing rate of the FFF-VMAT linac decreased below 95%.

## Discussion

The result of this study show that the average HI for the cone-based linac was lower than those for the FFF-VMAT linac and tomotherapy because of the radiation of the cone-based linac was homogeneous. As a result of no dose-intensity control within the radiation field of cone-based linac, there were no significant changes for a cone treatment at a distance difference of only 5 mm distance to brainstem.

Because the radiation field of the cone-based linac was similar to the size of the tumors, this modality yielded the highest CGIc and CIPaddick. Unlike the cone-based linac, tomotherapy uses a constant jaw size, leading to a “ramp-up” effect, which causes normal tissues surrounding the tumor in the S–I direction to receive a higher dose [23].

The use of a cone in the cone-based linac minimized the dose divergence, resulting in the cone-based linac having the best CGIg and GIPaddick among the three treatment modalities. When the tumor volume was very small, the jaw used in tomotherapy limited the dispersion range of the low-dose region, causing the CGIg to be higher than that for the FFF-VMAT linac. As the tumor volume increased, the dose on the brainstem increased.

The research of Yip et al. [12] indicated that, regardless of the conformity and gradient, the cone-based linac performed better than tomotherapy for tumors with a regular shape; this finding is consistent with our results. However, Soisson et al. [24] reported that excellent levels of tumor conformity were achieved using tomotherapy. Nevertheless, we obtained a similar result with respect to the overall evaluation of the CGI. Soisson et al. did not provide a detailed comparison between the doses of the FFF-VMAT linac and tomotherapy for SRT, whereas we showed that, regardless of the tumor size and distance from the brainstem, the conformity of the FFF-VMAT linac was always better than that of tomotherapy. The FFF-VMAT linac also had a steeper gradient than tomotherapy for larger tumors, whereas tomotherapy exhibited a steeper gradient for smaller tumors.

It will result in the dose accumulation to the borders of 18 mm and 28 mm diameter PTV located 1 mm from brainstem if we try to lower the dose of brainstem for FFF-VMAT linac and tomotherapy with intensity modulated field. In this situation, if we take into account the tumor coverage, it may increase the dose closed to the borders of PTV. For cone-based linac without intensity modulated field, the dose in the PTV will be more uniform and the dose of brainstem will be reduced because of high dose gradient. According to the AAPM TG-142 report, a deviation of 1 mm is allowed in the rotational center of the couch and gantry of the linear accelerator used for SRS or SRT, and the repeatability of the MLC is required [25]. These deviations could have affected the gamma analysis results for regions with a steep dose gradient. O’Connor et al. [26] reported that errors in the position of the MLC significantly affected the results of gamma analysis; for example, with the 3% 1-mm criteria as the gamma analysis passing metric, a 0.8 mm deviation in the position of the MLC reduced the gamma passing rates of square-field rotating beams with field sizes of 16 and 40 mm by 5.7% and 4.5%, respectively. In the continuous delivery of VMAT, the position of MLC at each control point must match the gantry’s speed and dose rate, and the MLC position in this continuous process will also be affected by gravity and the speed of MLC. Since the setting of MLC’s tolerance table in machine’s setting is 1 mm, it means that the machine will keep delivery as the MLC position error is less than 1 mm at each control point in the process of delivery, and that the 1 mm tolerance may affect the measurement results, especially in the small field and high-dose gradient regions [27, 28]. Therefore the more mechanical error variable, the more likely to affect the Gamma pass rate.

The treatment time of each technique is also the focus of our concern. In our study, the longest treatment time required for the cone-based linac, FFF-VMAT linac and Tomotherapy were 830, 679 and 728 s, respectively. The above time did not include the time of image registration and confirmation. For cone-based linac and FFF-VMAT linac techniques using the non-coplanar angles, the number of beams that need to rotate the angle of the couch which can affects the time required for treatment.

## Conclusion

As a result of the use of spherical tumors, we could not be affected by the shape of the tumor and clearly understand the differences of the dose characteristics between three modalities. Among the three treatment modalities studied, the cone-based linac had the best conformity and dose gradient for tumors of all sizes and locations. According to our results, if a critical organ, such as the brainstem, is located near the tumor and the situation requires a steep dose gradient, the cone-based linac should be used for SRT therapy. Since the steep dose gradient of the cone-based linac is obvious, we should also consider using a high dose gradient of the cone base to treat slightly irregular tumor and protect the critical organs or normal brain. The dose conformity of the FFF-VMAT linac for tumors of all sizes and positions was better than that of tomotherapy. The dose gradient of the FFF-VMAT linac for large tumors (28 mm in diameter) was better than that of tomotherapy, whereas tomotherapy had a better dose gradient than the FFF-VMAT linac for small tumors (8 mm in diameter). The cone-based linac had the smallest 50% and 30% dose profile widths in the R–L and S–I directions among the three modalities, with the exception of the 30% dose profile width for 28 mm tumors in the S–I direction, where tomotherapy and the cone-based linac produced similar results. On one hand, the 30% dose profile widths of the FFF-VMAT linac in the R–L direction for all tumor volumes were smaller than those of tomotherapy. Therefore, for protecting normal tissues located superior and inferior to the tumor, we can consider using tomotherapy or Cone base with couch at 0 ° for treatment. The TLD and EBT3 measurement results indicate that all three SRT treatment modalities achieved accurate doses. However, the FFF-VMAT and cone-based linacs may have produced dose deviations in regions with steep gradients on the borders of the tumor because of the effects of mechanical factors.

## Abbreviations

3D: Three-dimensional
CGI: Conformity/gradient index
CGI_c_: Conformity index
CGI_g_: Gradient index
CI_Paddick_: Paddick conformity index
CT: Computed-tomography
FFF: Flattening filter-free
GI_Paddick_: Paddick gradient index
HI: Homogeneity index
Linac: Linear accelerator
MLCs: Multileaf collimators
R–L: Right–left
S–I: Superior–inferior
SRS: Stereotactic radiosurgery
SRT: Stereotactic radiotherapy
TLD: Thermoluminescent dosimeter
VMAT: Volumetric modulated arc therapy
